# The Preventive Treatment of Puerperal Fever

**Published:** 1878-04-20

**Authors:** S. E. Robinson

**Affiliations:** West Union, Iowa


### THE PREVENTIVE TREATMENT OF PUERPERAL FEVER.

By S. E. Robinson, M.D., of West Union, Iowa.

In No. 20, Vol. xxxvii, *Medical and Surgical Reporter*, I find a notice, under "Periscope," of "Local and Preventive Treatment of Puerperal Fever," from Dr. Fritsch. I have for sometime been surprised to see little under that head in our medical periodicals. We should have had many articles from the pens of the best men in the profession, calling attention to the prophylaxis in this serious disease.

A few years since, in the discussion of a paper on puerperal fever, presented to the Iowa State Medical Society, Professor Cleaver, of Keokuk, remarked that he could say very little as to the best methods of treatment in this disease, for the reason that he had not seen a case in his own practice for a number of years, or at least, not when his directions had been followed in the treatment of the post-partum period. I was surprised—I may say astonished—to hear that from a man of his standing and extensive practice in midwifery; for, practicing as I do, in a small town, with decidedly healthful surroundings, and doing a not remarkably large obstetric business, I was in the habit of seeing several cases annually, and I was confident that I used at least ordinary care and skill in the management of my patients.

Dr. Cleaver went on to say that he uniformly directed a copious vaginal injection of carbolized water, two, three or four times a day, for several days following delivery, and that in no case, where his directions were obeyed, had he seen a case of puerperal fever.

I may not have reported here just the doctor's language, but have given the substance of his remark. I do not remember just how he prepared the fluid for injection, just the quantity of carbolic acid to the pint of water (he never used less than a pint at each operation, I think), but ever since I got the idea from him, I have used it in practice with perfect success, as have several of my professional neighbors and friends, and I do not doubt many others are doing the same elsewhere.

Professor Cleaver believed the disease was occasioned almost entirely by the absorption of septic matter from the decomposition of retained material in the vagina. Blood and shreds of membrane retained in the uterus would be more likely to be expelled, or drain into the vagina, than they would to escape from the vagina before decompositon and leave it free and clean; consequently, the thorough washing of the vaginal mucous membrane, with plenty of warm water, is the thing of greatest importance. Then, to provide against a possible want of thoroughness in the operation, it is well to add a disinfectant. My practice is to prescribe equal parts of carbolic acid and glycerine mixed (the acid is more readily and perfectly soluble in water if at first dissolved by glycerine); of this, I direct a teaspoonful to each pint of warm water, or Castile soap and water, to be used as a vaginal douche, or injection, not less than a pint to be used at each operation, and to be repeated, in ordinary cases, three times a day, for the first two or three days, then twice daily for three or four days longer. I seldom find a patient who is not quite willing to follow instructions in this matter, the comfort and relief from "after pains" being sufficient inducement, even though there be no reason to fear any fever or other complications.

In cases where the labor has been tedious, or where turning, forceps, or any operation has been performed, I direct that the injection be repeated for the first three days every four or six hours, using sufficient quantity that it shall flow from the vagina free from any stain of blood, clear. Should the lochia be suppressed, I still advise the copious injection; this, however, seldom occurs, though the nurse may not discover much flow, for reason of the discharge being carried away by the douche. In a few cases that had no tediousness or complication, I have directed simply Castile soap and water, and with good results.

In a few cases, the family having no convenience for using the vaginal wash, having neglected it after being advised to procure a syringe, etc., I may say a Mattison, Davidson, or any syringe capable of giving a continuous stream, or using a quantity of fluid without repeated introductions, and a tin wash dish, is all that is *necessary*, though a bed pan is quite a convenience. Then, sometimes, I am called two or three days after delivery to find my patient has had chill, followed by fever, abdominal tenderness, arrest of the lochial discharge, and all the symptoms of the beginning of puerperal fever. My treatment then is, to insist upon the immediate use of the vaginal douche; sometimes direct a saline cathartic, and if there be any reason to suspect malarial complications, give a few doses of quinine, and if there be no malaria, the quinine does no harm; it stimulates more perfect and rapid involution, adds to the nervous force of the patient, and in that way helps to eliminate, if it does not neutralize, poison in the blood.

I can now say, with Professor Cleaver, that "I have not had a case of puerperal fever for several years." I have not used the precaution advised by Dr. Fritsch, of disinfecting the hands, but I approach my cases always, I think, with clean hands, and should not object to the use of disinfectants, brushed around and under the nails, but have not found it necessary.—*Med. Rep.*

